# Supplementary material for: Electron stochastic acceleration in laboratory-produced kinetic turbulent plasmas
Source: Nat Commun. 2024 Jul 13;15:5897. doi: 10.1038/s41467-024-50085-7 (PMC11246523; doi:10.1038/s41467-024-50085-7)
Supplement: Supplementary file 1 — Supplementary Information [file 41467_2024_50085_MOESM1_ESM.pdf]

Electron stochastic acceleration in laboratory-produced kinetic turbulent plasmas

Dawei Yuan[1,2*], Zhu Lei[3,4,5*], Huigang Wei[1], Zhe Zhang[6,7,8], Jiayong Zhong[2,9], Yifei Li[6], Yongli Ping[9], Yihang Zhang[6], Yutong Li[6,7,8,10,†], Feilu Wang[1,11], Guiyun Liang[1,2], Bin Qiao[4,5,12 †], Changbo Fu[13], Huiya Liu[14], Panzheng Zhang[14], Jianqiang Zhu[14], Gang Zhao[1,11,†] and Jie Zhang[7,15,16,17†]

[1]Key Laboratory of Optical Astronomy, National Astronomical Observatories, Chinese Academy of Sciences, 100101 Beijing, P. R. China

[2]Institute of Frontiers in Astronomy and Astrophysics of Beijing Normal University, 100875 Beijing, P. R. China

[3]Institute of Applied Physics and Computational Mathematics, 100088 Beijing, R. P. China

[4]School of Physics, Peking University, 100871 Beijing, P. R. China

[5]Center for Applied Physics and Technology, Peking University, 100871 Beijing, P. R. China

[6]National Laboratory for Condensed Matter Physics, Institute of Physics, Chinese Academy of Sciences, 100190 Beijing, P. R. China

[7]Collaborative Innovation Center of IFSA, Shanghai Jiao Tong University, 200240 Shanghai, P. R. China

[8]Songshan Lake Materials Laboratory, Dongguan, 523808 Guangdong, P. R. China

[9]Department of Astronomy, Beijing Normal University, 100875 Beijing, P. R. China

[10]School of Physical Sciences, University of Chinese Academy of Sciences, 100049 Beijing, P. R. China

[11]School of Astronomy and Space Science, University of Chinese Academy of Sciences, 101408 Beijing, P. R. China

[12]Frontiers Science Center for Nano-optoelectronic, Peking University, 100094 Beijing, P. R. China

[13]Key Laboratory of Nuclear Physics and Ion-Beam Application (MoE), Institute of

Modern Physics, Fudan University, 200433 Shanghai, P. R. China

[14]National Laboratory on High Power Laser and Physics, Shanghai Institute of Optics and Fine Mechanics, Chinese Academy of Sciences, 201800 Shanghai, P. R. China

[15]Tsung-Dao Lee Institute, Shanghai Jiao Tong University, 201210 Shanghai, P. R. China

[16]Key Laboratory for Laser Plasmas (MoE), Shanghai Jiao Tong University, 200240 Shanghai, P. R. China

[17]Department of Physics and Astronomy, Shanghai Jiao Tong University, 200240 Shanghai, P. R. China

*These authors contributed equally: Dawei Yuan and Zhu Lei

† Email: ytli@iphy.ac.cn;

bqiao@pku.edu.cn;

gzhao@bao.ac.cn;

jzhang1@sjtu.edu.cn

Supplementary Notes

Supplementary Note I. The Coulomb collisions

Interpenetrating flows are commonly utilized as a testbed for studying various astrophysical phenomena, including collsionless shocks, kinetic turbulence, and particle acceleration. The presence of Coulomb collisions during the interaction of both flows plays a crucial role in the physical evolution process. The collisions, which occur between different species such as electrons and ions, can be referred as inter-flow collisions in interpenetrating flows and intra-flow collisions within the same flow. Here we will estimate the Coulomb collisions under our experimental conditions.

Inter-flow collisions are mainly determined by the flow velocity. Electron-electron, ion-ion, and ion-electron collisions should be considered in our experiments. First, the collisions between electrons are frequent, due to the thermal velocity larger than the flow velocity (V_{the}/V_{flow} ~ 6). Therefore, electron population can be characterized by a thermalized background in our experiments. While ion-ion collisions and ion-electron collisions should be addressed.

The mean free path (MFP) for ion-ion collisions in interpenetrating flows can be expressed as[1],

$$\lambda_{i\text{-}i}^{inter}[\mathrm{cm}] \sim 670A_i^4(V_r[1000\mathrm{km/s}])^4/((Z_i^4 2A_i)^2 n_i[10^{19}\mathrm{cm}^{-3}]\ln\Lambda). \qquad (1)$$

For the parameters of our experiments, the relative velocity $V_r = 2V_{flow}$ = 3000 km s^{-1}, the ion density $n_i = n_e/Z_i = 0.5\times10^{19}$ cm^{-3}, and the Coulomb logarithm $\ln\Lambda \sim 8.5$, we have $\lambda_{i\text{-}i}^{inter}$ ~ 200 cm, largely exceeds our target size (L = 3.2 mm).

The MFP for ion-electron collisions in interpenetrating flows can be expressed as[2],

$$\lambda_{i\text{-}e}^{inter}[\mathrm{cm}] \sim 6\times10^{-3}A_iV_r[1000\mathrm{km/s}]\ T_e^{3/2}(\mathrm{eV})/((Z_i^2 n_e[10^{19}\mathrm{cm}^{-3}]\ln\Lambda), \qquad (2)$$

which yields $\lambda_{i\text{-}e}^{inter}$ ~ 31 cm, also larger than L.

From above estimation, one can expect that both counter-streaming plasma flows can freely interpenetrate ($V_{flow} > V_{thi}$) in a thermalized electron background, allowing us to study turbulence originating from ion-driven-Weibel instability and electron acceleration.

Intra-flow collisions are primarily influenced by the temperature of the plasma flow (T_i = T_e = T). This is due to the supersonic nature of the laser-produced plasma flow, resulting in significantly lower thermal energy compared to kinetic energy. As a result, the Coulomb collision cross-section scales inversely with the square of the temperature ($1/T^2$).

The corresponding MFP for same species is written as[1],

$$\lambda_{\alpha\text{-}\alpha}{}^{intra}[\mathrm{cm}] \sim 0.5(T_\alpha[0.5\mathrm{keV}])^2/(Z_\alpha{}^4 n_\alpha[10^{19}\mathrm{cm}^{-3}]\ln\Lambda), \quad (3)$$

where α stands for the species (electron and ion). We have $\lambda_{i\text{-}i}{}^{intra} \sim 1 \times 10^{-2}$ cm and $\lambda_{e\text{-}e}{}^{intra} \sim 10^{-3}$ cm, much smaller than the expanding plasma size (3 mm × 2 mm).

One can see that intra-flow collisions are frequence, purely hydrodynamical flow. These intra-flow collisions can contribute to the thermal equilibrium within the flow. Recent works[3,4] have shown that the Weibel instability can develop fully in the current experimental conditions. It suggests that the intra-flow collisions play a minimal role in the overall evolution.

All the key parameters of Coulomb collisions in the experiments have been summarized in Supplementary Table 1. One can see that the turbulent plasmas originating from the nonlinear Weibel instability are easily produced in the experimental conditions.

Supplementary Table 1 | The mean free path (MFP) for Coulomb collisions between the different species in the experiments.

Collision type	ion-ion	ion-electron	electron-electron
MFP in inter-flows	~ 200 cm	~ 31 cm	~ 10^{-3} cm
MFP in intra-flow	~10^{-2} cm	~ 5×10^{-2} cm	~ 10^{-3} cm

Here we estimate above parameters using the plasma flow conditions including flow velocity V_{flow} = 1.5×10^8 cm s^{-1}, electron density $n_e = Z_i n_i \approx 10^{19}$ cm^{-3}, and plasma temperature $T_e = T_i \approx 600$ eV.

Supplementary Note II. Thermal electrons from the single plasma flow

In order to distinguish between accelerated electrons in the turbulent region and thermal electrons in the single plasma flow, it is essential to compare the electron

spectrum in both the single-flow and interpenetrating-flow scenarios. This comparison allows us to identify the difference between non-thermal and thermal spectra. Our experimental setup involves the electron magnetic spectrometer (EMS) being positioned at a 70-degree angle respective to the x-axis, as illustrated in Supplementary Fig. 1. As a result, we have obtained two different thermal electron spectra from different flows: one from a single flow moving in the +x direction, and the other from a single flow moving in the -x direction. Analysis of the data revealed significant differences between the two electron spectra. While both spectra exhibit characteristics of Maxwell distributions, such as shape, the number of thermal electrons and cut-off energy vary greatly. These differences are influenced by the observation angle of the ESM. When the single flow propagates in the +x direction, only a small fraction of thermal electrons can be captured by the ESM. Conversely, most thermal electrons will bypass the ESM and enter directly into it. This observation underscores the importance of considering the angle and position of the ESM when analyzing electron spectra in plasma flows.

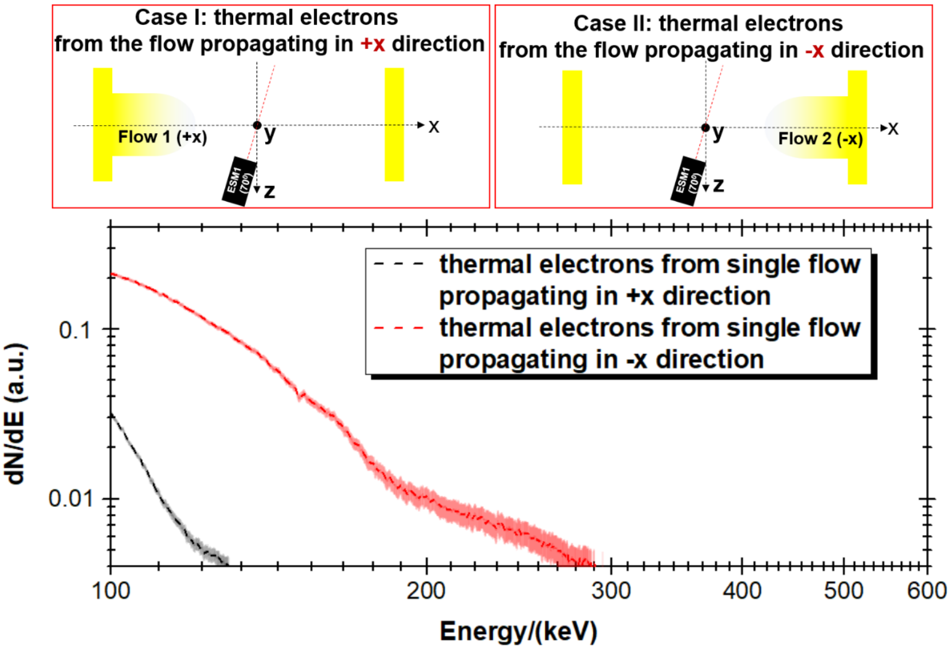

Supplementary Figure 1: Measurement of the thermal electrons from different single flows. One background (black) is obtained the flow propagating in the +x direction, and the other one (red) is obtained the flow propagating in the -x direction. The number density of electrons is normalized to the maximum value. The shaded region corresponds to the error bar of electron number ($\Delta n/n$ ~20%, where Δn is

electron number error mainly caused by sensitivity of image plate).

Supplementary Note III. Non-thermal electrons from the turbulent plasmas

The measured non-thermal electrons in our experiment are free electrons that escape the turbulent region (interpenetrating flows) and enter the EMS. The electron spectra at three observation angles are measured by three separate ESMs, as shown in Supplementary Fig. 2. It is important to note that the measured spectral shape is completely different from that observed in a single flow. Furthermore, we can observe that non-thermal electrons exhibit the same power-law distribution E^{-3}, with a similar number of non-thermal electrons present. These crucial findings suggest that electron acceleration is isotropic, in contrast to previous studies which indicated that accelerated electrons move in specific directions [1,5,6,7]. It is believed that stochastic acceleration is the primary contributor to the generation of these non-thermal electrons. The corresponding simulations reveal that as the magnetic island-like structures form within the turbulence, thermal electrons are energized and experience a net gain after frequent collisions with these islands. The electron spectral index obtained in simulations ultimately aligns well with the measurements.

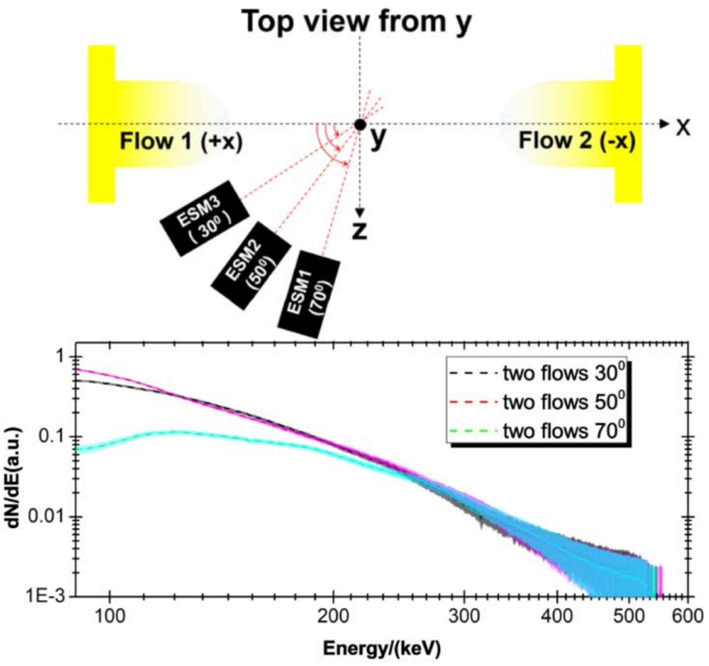

Supplementary Figure 2: Angular distribution of non-thermal electron energy spectra from the turbulence. The number density of electrons is normalized to the maximum value. The shaded region

corresponds to the error bar of electron number ($\Delta n/n$ ~20%, where Δn is electron number error mainly caused by sensitivity of image plate).

Supplementary Note IV. The linear growth rate of Weibel instability

In our experiments, the linear growth rate of Weibel instability is derived from the dispersion relation of plasma instabilities for unmagnetized interpenetrating flows (±x), which has been extensively explored in many previous papers [8,9]. Specifically, here we focus on the ion-driven Weibel instability, which grows perpendicularly to the flow propagation direction. In this scenario, the electrons have established a thermalized background with the conditions of $V_{the} >> V_{flow} >> V_{thi}$. The simplified dispersion relation for Webel instability can be expressed as follows:[3]

$$k_y^2c^2 + \omega_{pi}^2/(1 + |k_y|/\Gamma\sqrt{2T_e/\pi m_e}) +$$

$$\omega_{pi}^2[G_1(\Gamma^2Am_p/(2k_y^2T_i)) - k_y^2V_{flow}^2/\Gamma^2G_2(\Gamma^2Am_p/(2k_y^2T_i)] = 0. \qquad (4)$$

Here c represents the speed of light, k_y is the most unstable wave number perpendicular to the flow direction, V_{flow} is the flow velocity, A is the atomic number, m_p is the proton mass, Γ is the linear growth rate. Additionally, G_1 and G_2 are the dimensionless functions defined for x > 0 as

$$G_1(y) = 1/\sqrt{\pi}\int y e^{-x2}/(x^2+y)dx$$

$$G_2(y) = 2y/\sqrt{\pi}\int x^2 e^{-x2}/(x^2+y)dx \qquad (5)$$

Taking experimental parameters into above dispersion equation, we obtain the relationship between the linear growth rate Γ and the wave number k_y, as shown in the Supplementary Figure 3. The most unstable mode of Weibel instability observed in our experiments is $k_y \sim 1/(L_{spacing}) = \omega_{pi}/c$, that is to say $k_yc/\omega_{pi} \sim 1$. Based on data presented in the Supplementary Fig.3(a) and 3(b), we can obtain the linear growth rate is $\Gamma \sim 0.1V_{flow}/c\omega_{pi} \sim 1.5 \times 10^9$ s^{-1}.

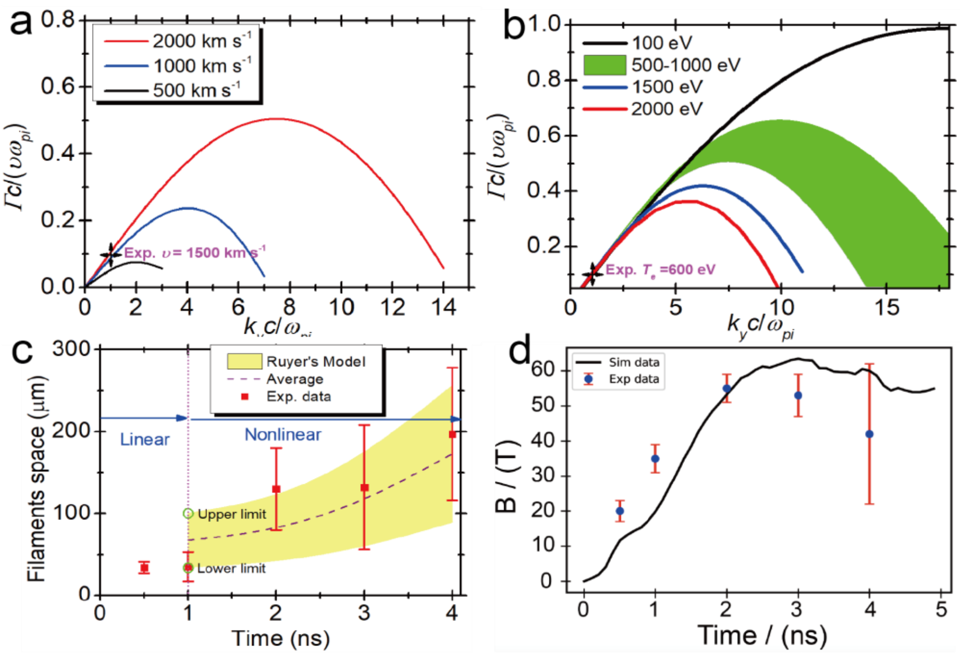

Supplementary Figure 3: The growth of Weibel instability derived from experiment and theory. The linear growth rate (normalized to $\upsilon/c\times\omega_{pi}$) of the Weibel instability versus transverse wavelength (normalized to ω_{pi}/c), which is derived from the electromagnetic dispersion relation. It shows that this robust WI grows over a wide range of flow velocity (a) and temperature (b). **a,** The maximum linear growth $\Gamma c/(\upsilon\omega_{pi})$ via the flow velocity of 500 km s^{-1} (black), 1000 km s^{-1} (blue) and 2000 km s^{-1} (red), where the flow density of 1×10^{19} cm^{-3} is used from the Nomarski data. **b,** The maximum linear growth $\Gamma c/(\upsilon\omega_{pi})$ via the flow temperature of 100 eV (black), 500-1000 eV (green), 1500 eV (blue) and 2000 eV (red), where the flow velocity of 1.5 $\times10^{8}$ km s^{-1} is used from the streaked interferometry. **c,** Comparison of the mean filaments space between measurement and theory. The experimental filament spacing value is calculated with statistical analysis method. The error bar is the fullwidth at half-maximum of the spacing distribution function. The theoretical value is addressed with analytical model, where the time-dependent transverse wavelength is expressed as $\lambda_y(t) = \lambda_y(t_*)[1+(t-t_*)^2/\tau_0^2]$. Here, $t_*\approx1.0$ ns is the end time of the linear stage, $\tau_0 = 2\pi[\lambda(t_*)c/\omega_{pi}]^{1/2}(4m_i/Zm_e)^{1/4}/\upsilon = 2.6$ ns is the typical coalescence time and $\lambda_y(t_*) = 40\text{-}100$ μm is the initial space value. **d,** The evolution of Weibel magnetic field strength obtained from measurements and simulations. One can see that the magnetic fields initial grow quickly in the form of exponent ~ $e^{\Gamma t}$, where Γ is the linear growth of Weibel instability. And then, the magnetic fields tend to saturation, suggesting that conversion of kinetic energy of plasma flow to magnetic energy reaches equilibrium.

Supplementary Note V. The comparison of typical parameters in laboratory and

supernova

It is well-known that using laser-produced interpenetrating flows can investigate the astrophysical shock formation and particle acceleration under the scaling-law. Recently, experiment performed at NIF has shown that Weibel-mediated shock have the ability to accelerate electrons to relativistic velocities through 1st Fermi acceleration[1]. As illustrated in Supplementary Table 2, our experimental plasmas and supernova plasmas share a number of dimensionless parameters, such as Peclet number (P_e >>1), Reynolds number (R_e >>1), and magnetic Reynolds number (R_M >>1). The similarity of these dimensionless parameters indicates that the dominated physical process in both systems is similar. This similarity allows our study to investigate acritical transition period in supernova explosion[10,11] where kinetic turbulences arising from Weibel instability have been generated but the shock has not yet formed. These finding sheds light on the complex dynamics at play during this crucial phase of supernova.

Supplementary Table 2 | The comparison of typical parameters in laboratory and supernova.

Parameters	Laboratory plasmas	Supernova Plasmas (SN 1987)[12]
Magnetic field, B (G)	~ 10^5-10^6	~ 10^{-5}-10^{-4}
Number density, n_e (cm^{-3})	~ 10^{19}	~ 1
Flow velocity, V_{flow} (cm s^{-1})	~10^8	~ 10^8-10^9
Electron temperature, T_e (eV)	600	~ 1
Ion temperature, T_i (eV)	600	~ 1
Collisionality, ($L/\lambda_{i\text{-}i}$)	~ 10^{-3}	~ 10^{-2}-10^{-3}
Peclet number, P_e	~ 20	~ 10^7
Reynolds number, R_e	~ 10^2	~ 10^8
Magnetic Reynolds number, R_M	~ 10^5	~ 10^9

Note that the Peclet number $P_e = LV_{flow}/\chi$, χ is the thermal diffusivity taking into magnetic effect, the Renolds number $R_e = LV_{flow}/v$, v is the kinematic viscosity, the Magnetic Reynolds number $R_m = LV_{flow}/\eta$,

η is the magnetic resistivity.

Supplementary Note VI. The schematic diagram of optical diagnostics

As shown in Supplementary Figure 4, one probe beam propagating through the interested region is divided into three channels. One is for Faraday rotation to measure the self-generated magnetic fields, the second one is for Nomarski interferometer to measure the plasma density, and the third one is for shadowgraphy to measure the density gradient (here we measure the filaments). The parameters of expanding flow before colliding are measured by streaked camera where the probe works at long-pulse mode, while the characteristics of both interpenetrating flows are obtained by CCD where the probe works at short-pulse mode. The inset shows the schematic diagram of Faraday rotation measuring the self-generated magnetic fields via WI.

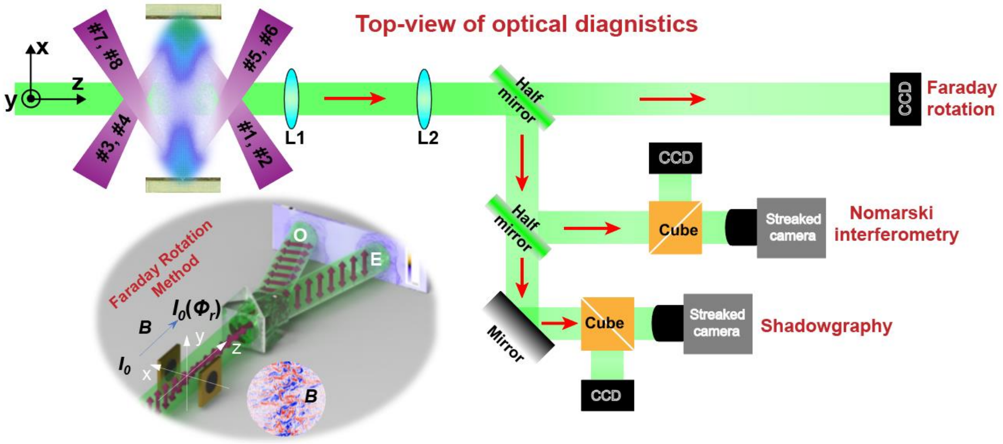

Supplementary Figure 4: The schematic diagram of optical diagnostics. The probe with initial polarization in y direction passing through the interaction region is divided into three channels: one is for Faraday rotation to measure the Weibel mediated magnetic fields, the second one is for Nomarski interferometry to give the plasma density, and the last one is for shadowgraphy to detect the current filaments. The whole imaging system consists of two stages with a magnification of 2.5x. The spatio-temporal resolutions are about 10-15 μm and 100 ps, respectively.

The raw images of the WI evolution obtained by shadowgraphy are shown in Supplementary Figure 5.

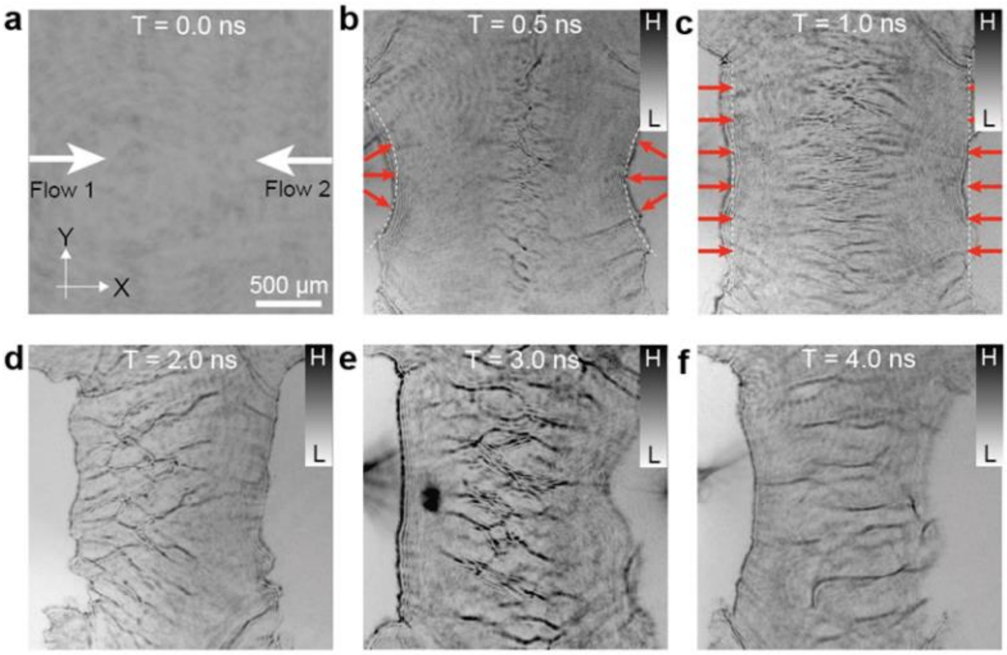

Supplementary Figure 5: The raw data of WI evolution obtained by shadwography. The probe flux is modulated by the plasma density, reflecting the redistribution of plasma density information. **a**, Image displaying the probe flux shows a very uniform distribution before shooting, with no hint of filamentary structures. **b-c**, Local ordered filaments form at the midplane and stretch along the flow direction with both flow interpenetration. The plasma flow (white dotted lines) demonstrates a transition from spherical shape (**b**) to quasi-planar shape (**c**) (red arrows). Such a transverse change favor to the WI. **d-f**, Filaments become unstable, break, and coalesce each other, stepping into the nonlinear stage.

Supplementary Note VII. The evolution of fluid dynamics

The typically initial parameters of plasma flow are obtained by the experiments and corresponding simulations. The plasma flow density distribution (Supplementary Figure 6(a)) is measured using the Nomarski interferometer with the charge coupled devices (CCD) and the plasma flow velocity (Supplementary Figure 6(c)) is measured using the Nomarski interferometer with the streaked camera. The plasma flow temperature (Supplementary Figure 6(b)) is inferred from the quasi-isothermal expanding theory by fitting the measured density profile. These parameters are also derived from 3D FLASH simulations, as shown in Supplementary Figure 6(d) to 6(f). One can see that both matches well.

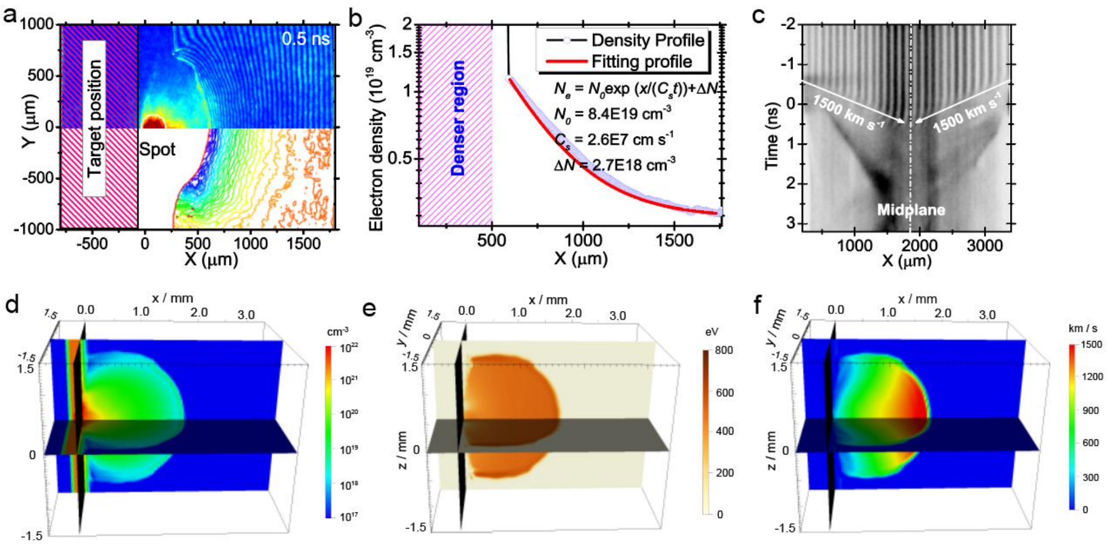

Supplementary Figure 6: The parameters of plasma-flow characterized by experiments and simulations. **a-c,** The electron density, plasma temperature and flow velocity obtained by optical diagnostics. The density of plasma plume is in the region of 0.5-1×10^{19} cm-3. The ion sound velocity is about 2.6×10^{7} cm s^{-1} by fitting the density profile basing the quasi-isothermal expanding, corresponding to the maximum flow temperature of 600 eV. The velocity of plasma almost keeps at 1.5×10^{8} cm s^{-1}. **d-f,** The corresponding parameters of single flow obtained from 3D FLASH simulation, well agreement with the experimental measurement.

As shown in the Supplementary Figure 7, obtained by the streaked optical diagnostics, we have measured the evolution of the single flow free expansion (0-18 ns) and interpenetrating flow interactions (0-8 ns). Obviously, these plasma flows are sufficient to drive the evolution of the Weibel instability and the formation of the turbulent plasmas.

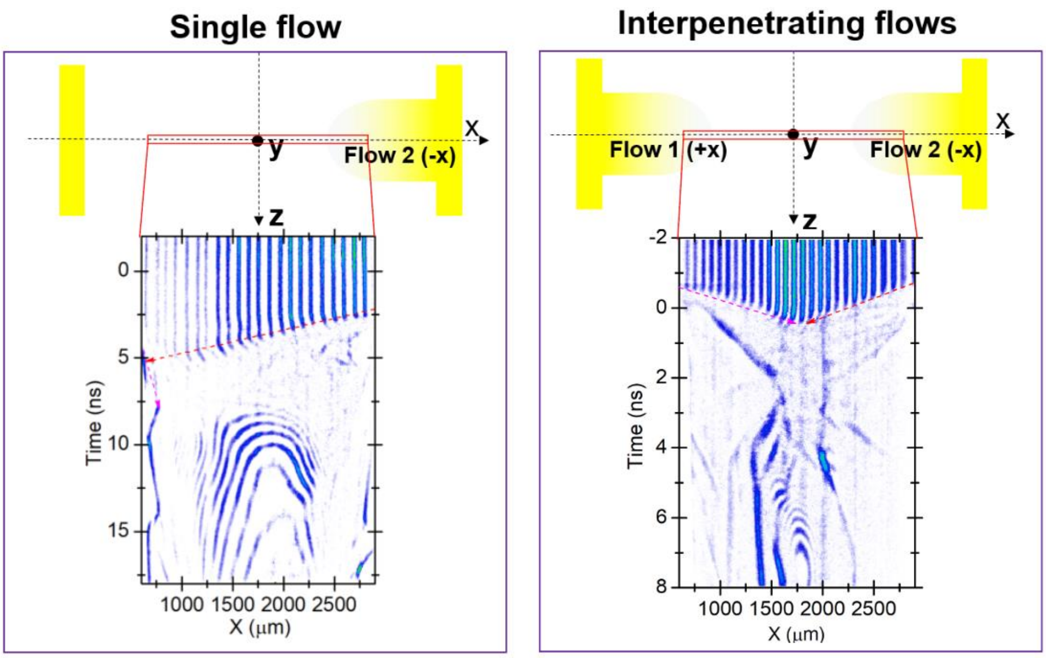

Supplementary Figure 7: The comparison of the plasma evolution between the single flow and interpenetrating flows. Interferometry coupled with streaked camera provide measurement of the flow velocity and lifetime for the single flow (left) and two interpenetrating flows (right).

For later stage of the evolution of fluid dynamics, Supplementary Figure 8 show the distribution of mass density from 15 ns to 25 ns. the plasma flow has not disappeared even at 25 ns. We also plot the density and velocity temporal profiles of a single flow at the midplane region obtained from the RMHD simulation (Supplementary Figure 9), we can observe that despite a decrease in velocity, the plasma flow remains present throughout. In the early stage, predominantly low-density high-speed plasma flow is observed, whereas in the later stage, it transitions to low-speed high-density plasma.

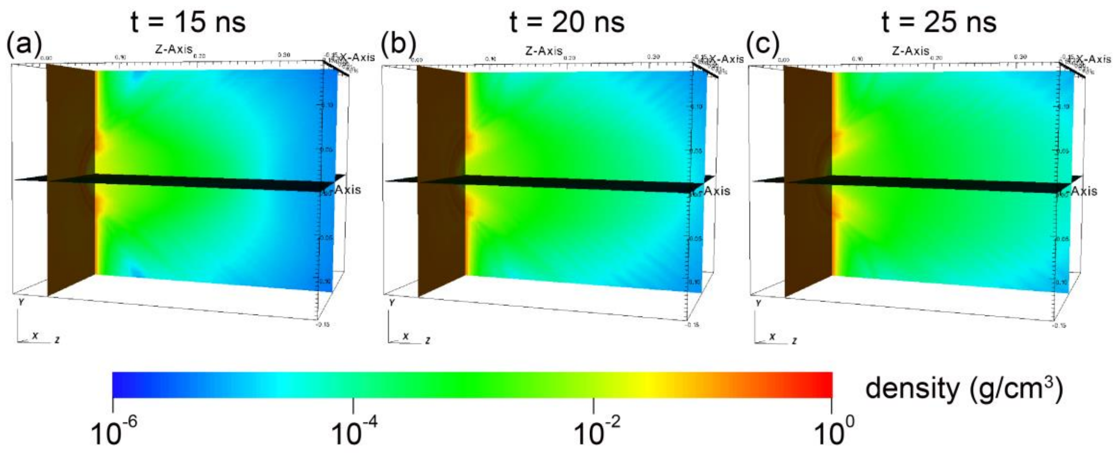

Supplementary Figure 8: Density distribution of RMHD simulation results at late stage. Panels (a) to (c) show the density distribution of 3D-RMHD simulation for a single flow at t = 15, 20, 25 ns respectively.

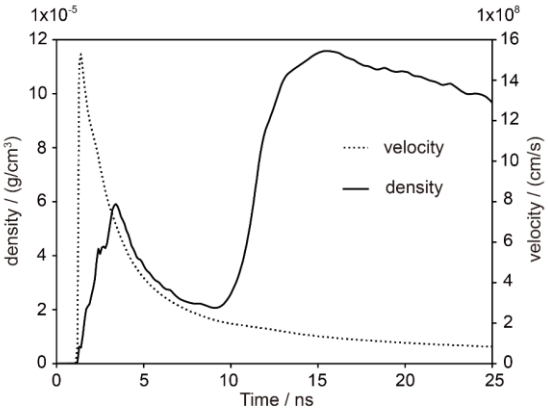

Supplementary Figure 9: RMHD simulation results. It depicts the temporal profiles of density (solid line) and velocity (dashed line) for a singular flow at the midplane region.

Supplementary Note VIII. More example of trajectory of accelerated particles

Similar to the trajectory of accelerated particle in Fig. 4, we present additional trajectory plots illustrating the stochastic acceleration of particles. Their evolutionary trajectories exhibit similar characteristics. In the early stage, the particles are trapped inside the magnetic island without significant change in energy. In the late stage, rapid acceleration occurs as the particle undergoes multiple collisions with magnetic islands, leading to a substantial energy boost from 20 keV to 100 keV, thereby transforming them into a superthermal particles.

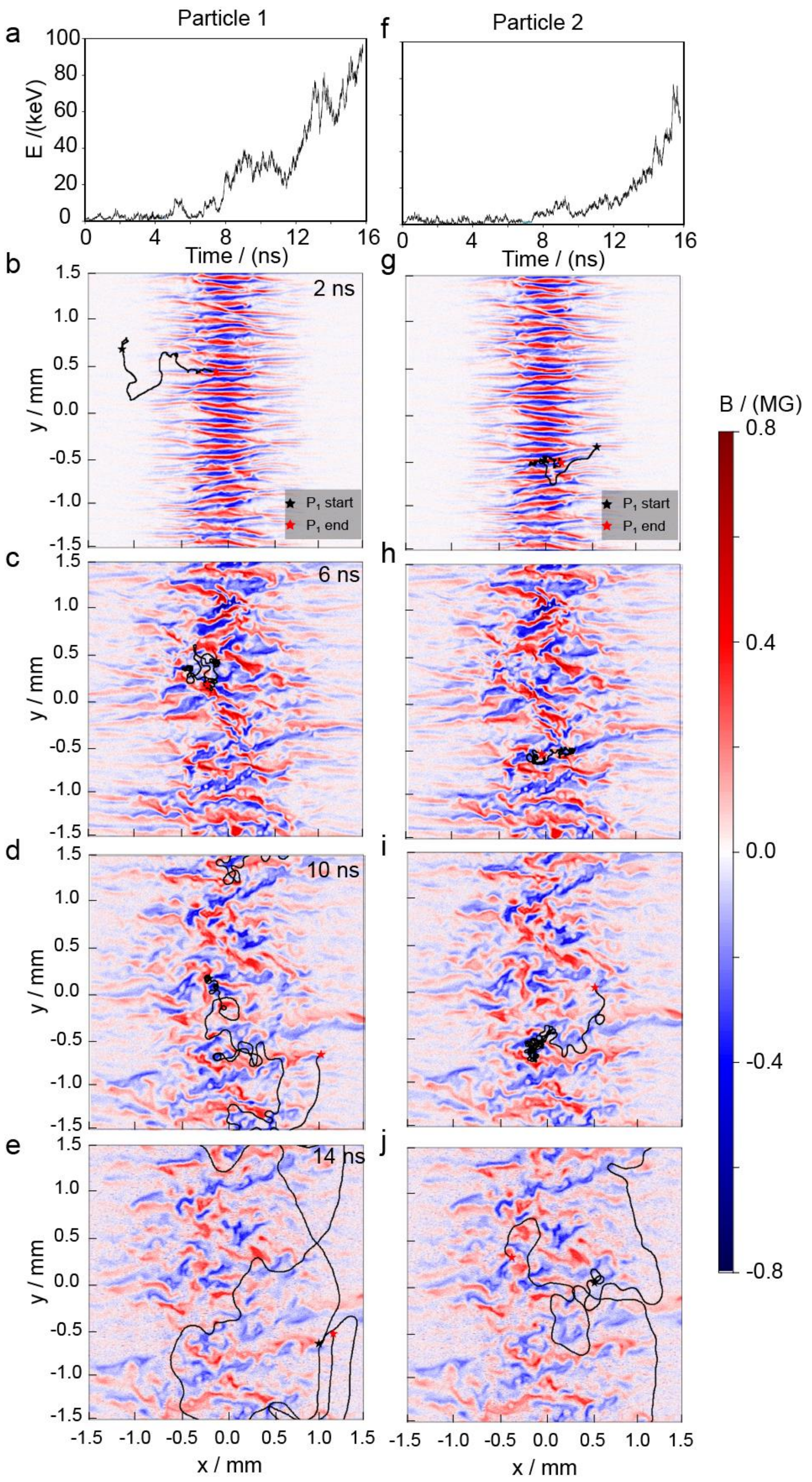

Supplementary Figure 10: The typical particle trajectories in Weibel turbulent plasmas. The top panels (**a** and **f**) illustrate the kinetic energy histories of two selected electrons over time. The left panels (**b** to **e**) display the trajectories of particle 1 at different time intervals, while the right panels (**g** to **j**) depict the trajectories of particle 2. In each panel, the black stars indicate the initial positions of the particles, while the red stars indicate their positions at the end of each interval.

Supplementary References

1. Fiuza, F. et al. Electron acceleration in laboratory-produced turbulent collisionless shocks. *Nat. Phys.* **16**, 916–920 (2020).

2. Zakharov, Y. P. et al. Comment on “Studying astrophysical collisionless shocks with counterstreaming plasmas from high power lasers”. *High Energy Density Phys.* **8**, 329-330 (2012).

3. Huntington, C. M. et al. Observation of magnetic field generation via the Weibel instability in interpenetrating plasma flows. *Nat. Phys.* **11**, 173-176 (2015).

4. Fox, W. et al. Filamentation instability of counterstreaming laser-driven plasmas. *Phys. Rev. Lett.* **111**, 225002 (2013).

5. Yao, W. et al. Laboratory evidence for proton energization by collisionless shock surfing. *Nat. Phys.* **17**, 1177-1182 (2021).

6. Ping, Y. L. et al. Turbulent magnetic reconnection generated by intense lasers. *Nat. Phys.* **19**, 263–270 (2023).

7. Chien, A. et al. Non-thermal electron acceleration from magnetically driven reconnection in a laboratory plasma. *Nat. Phys.* **19**, 254–262 (2023).

8. Liu, P. et al., Ion Kinetics and neutron generation associated with electromagnetic turbulence in laboratory-scale counterstreaming Plasma. *Phys. Rev. Lett.* **132**, 155103 (2024).

9. Kato, T. and Takabe, H. Electrostatic and electromagnetic instabilities associated with electrostatic shocks: Two-dimensional particle-in-cell simulation. *Phys. Plasmas* **17**, 032114 (2010).

10. McCray, R. and Fransson, C. The Remnant of Supernova 1987A. *Annu. Rev. Astron. Astrophys.* **54**, 19-52 (2016).

11. Bamba, A. et al. Small-scale structure of the SN 1006 shock with Chandra observation. *Astrophys. J.* **589**, 527 (2003).

12. Dyutov, D. et al. Similarity criteria for the laboratory simulation of supernova hydrodynamics. *Astrophys. J.* **518**, 821 (1999).
